# Interferences between breathing, experimental dyspnoea and bodily self-consciousness

**DOI:** 10.1038/s41598-017-11045-y

**Published:** 2017-08-30

**Authors:** Etienne Allard, Elisa Canzoneri, Dan Adler, Capucine Morélot-Panzini, Javier Bello-Ruiz, Bruno Herbelin, Olaf Blanke, Thomas Similowski

**Affiliations:** 1 Sorbonne Universités, UPMC Univ Paris 06, INSERM, UMRS1158 Neurophysiologie respiratoire expérimentale et clinique, Paris, France; 2 Laboratory of Cognitive Neuroscience, Center for Neuroprosthetics, Ecole Polytechnique Fédérale de Lausanne, Geneva, Switzerland; 3 Division of Pulmonary Diseases, Geneva University Hospital, Geneva, Switzerland; 4 AP-HP, Groupe Hospitalier Pitié-Salpêtrière Charles Foix, Service de Pneumologie et Réanimation Médicale (Département “R3S”), F-75013 Paris, France; 5 Department of Neurology, Geneva University Hospital, Geneva, Switzerland

## Abstract

Dyspnoea, a subjective experience of breathing discomfort, is a most distressing symptom. It implicates complex cortical networks that partially overlap with those underlying bodily self-consciousness, the experience that the body is one’s own within a given location (self-identification and self-location, respectively). Breathing as an interoceptive signal contributes to bodily self-consciousness: we predicted that inducing experimental dyspnoea would modify or disrupt this contribution. We also predicted that manipulating bodily self-consciousness with respiratory-visual stimulation would possibly attenuate dyspnoea. Twenty-five healthy volunteers were exposed to synchronous and asynchronous respiratory-visual illumination of an avatar during normal breathing and mechanically loaded breathing that elicited dyspnoea. During normal breathing, synchronous respiratory-visual stimulation induced illusory self-identification with the avatar and an illusory location of the subjects’ breathing towards the avatar. This did not occur when respiratory-visual stimulation was performed during dyspnoea-inducing loaded breathing. In this condition, the affective impact of dyspnoea was attenuated by respiratory-visual stimulation, particularly when asynchronous. This study replicates and reinforces previous studies about the integration of interoceptive and exteroceptive signals in the construction of bodily self-consciousness. It confirms the existence of interferences between experimental dyspnoea and cognitive functions. It suggests that respiratory-visual stimulation should be tested as a non-pharmacological approach of dyspnoea treatment.

## Introduction

Automatic medullary mechanisms ensure breathing continuity and adaptation to metabolic fluctuations in humans by sending neural commands to respiratory muscles that mobilise the rib cage and in turn inflate the lungs. This results in respiratory-related signals of various origins (upper airway, lungs and bronchi, the respiratory muscles, costovertebral joints) continuously being sent to the brain. Automatic breathing rarely reaches conscious awareness, because normal respiratory-related messages are considered redundant by the brain and extinguished, or “gated out”^1^. However, changes in respiratory pattern or respiratory mechanics may quickly lead to breathing awareness (“ungating”), pointing to an important role of respiratory-related afferents in self-consciousness. Because breathing also plays a role in sensing external stimuli such as ambient temperature and moisture and is intimately linked to the sense of smell^2, 3^, respiratory ungating occurs not only in response to interoceptive changes but also in response to exteroceptive changes (such as air cooling). Being at the crossroad of interoceptive and exteroceptive systems, breathing therefore contributes not just to own body perception, but also to the perception of body environment.

Recent work has suggested that interoceptive signals, including respiratory afferents, contribute to bodily self-consciousness (BSC)^4–6^. BSC encompasses the experience that the body is one’s own (self-identification) and the experience of being in a body with a given location within the environment (self-location), with a particular body-centred perspective (first person perspective)^7–10^. Recent research has revealed that BSC is based on multisensory brain mechanisms underlying the integration of tactile, proprioceptive, visual, and auditory cues, but also of interoceptive information^7, 11, 12^. Specifically, studies on multisensory perception have provided the groundwork for some of the fundamental laws of BSC^13^, based on the induction of experimentally controlled illusory states of BSC (i.e. the illusory feeling of an artificial body part as one’s own body). Such illusory states have been investigated by manipulating the spatiotemporal congruency of multisensory bodily signals (i.e. visuo-tactile stimulation; see below) during the full body illusion^14^ or related illusions^13, 15^. In the original full body illusion experiment, subjects saw their body from behind through a head-mounted display (HMD) while the investigator stroked their back. Subjects saw their body being stroked on the HMD either synchronously or asynchronously (with a 500 ms delay) with respect to the tactile input they received on their backs. In the synchronous illusion condition, participants mislocalised the touch to the virtual body and self-identified with it^14^. These subjective changes, as assessed by specific questionnaires, were concomitant with changes in implicit behavioural measures of self-location^14^. Changes in self-identification and self-location did not occur when the visual and the tactile inputs were presented asynchronously (control condition)^14^. Interestingly, it has been shown that similar changes in BSC can be obtained with visual feedback from interoceptive signals instead of tactile signals. Thus, a virtual hand^16^ or a virtual body^5^, flashing in synchrony with one’s own heartbeat induces changes in hand or body ownership, with participants self-identifying with the virtual body (or hand), whereas no change in BSC is observed when the flashing is provided asynchronously with the heartbeat or during other control conditions. Similarly, Adler and colleagues^4^ developed a respiratory-visual paradigm by adapting the previously described cardio-visual full body illusion to respiratory-visual stimulation. They reported that showing the participants a virtual body that was flashing synchronously with their breathing likewise induced subjective and behavioural changes in BSC. Again, no change was observed when the flashing was provided asynchronously with breathing. Most importantly, such changes were reflected in alterations of conscious breathing sensations such as breathing location (the perceived location of breathing) and breathing agency (the feeling of controlling the act of breathing for one’s body or the virtual body)^4^. These data suggest that interoceptive and exteroceptive signals jointly contribute to BSC. Moreover, recent data suggest that these signals are integrated in a distributed cortical network including the temporo-parietal cortex, premotor cortex, insula, posterior parietal cortex, and occipito-temporal cortex^17–19^. The default mode network has also been related to some aspect of self-processing^12, 20, 21^. Relevant to the present study, the insula is a key region associated with the processing of interoceptive signals^22, 23^. Moreover, recent work on BSC and other forms of conscious awareness have highlighted the role of the insula in integration of interoceptive and exteroceptive signals, in healthy subjects^6, 24^ and in neurological patients^25^.

Dyspnoea, defined as “a subjective experience of breathing discomfort that consists of qualitatively distinct sensations that vary in intensity”^26^, is a distressing and debilitating symptom common in respiratory diseases and many other disorders^26^. Dyspnoea is reported when the awareness of respiratory-related afferents (“somatic awareness”) is associated with negative feelings or emotions (“affective awareness”)^26^. Dyspnoea therefore combines, by definition, sensory and emotional aspects. Various types of experimental and clinical dyspnoea have been associated with the activation of cortical networks involving the supplementary motor area, the anterior cingula, the amygdala and also the insula^27–37^ and with deactivation of the default mode network^31^.

From the previously described contribution of breathing to BSC and from the commonalities between brain networks involved in BSC and dyspnoea, we hypothesised that experimentally inducing dyspnoea in healthy individuals would modify the effects of synchronous respiratory-visual stimulations. We reasoned that the bodily discomfort associated with dyspnoea could, by unbalancing the inputs that contribute to BSC, prevent synchronous respiratory-visual stimulations from producing their illusory effects. Indeed, with dyspnoea, the respiratory interoceptive information becomes intense and acquires a threatening nature. We also hypothesised that respiratory-visual stimulations could interfere with the evaluation of affective and cognitive breathing sensations. Such interferences may be related to various mechanisms, possibly combined, including attentional effects, emotional effects (that are intrinsic to dyspnoea by definition), and competition for cortical resources in line with previous descriptions of cross-talk between breathing and cognitive tasks in situations where respiratory-related cortical networks are activated^38, 39^. In the present experiment, we chose inspiratory threshold loading to induce dyspnoea because this paradigm (during which participants must overcome an inspiratory constraint to get air and then maintain their effort to continue breathing) has been associated with premotor and insular activations^31^ and may therefore interfere with BSC.

## Methods

### Participants

Twenty-five healthy subjects (20 female, median age [IQR]: 25.5 [23,32]) participated in the experiments. They all had normal or corrected to normal vision and no history of psychiatric, neurologic or respiratory diseases. The subjects received detailed information about the methods used in the experiment. They were informed of the respiratory purpose of the study, but were kept naive regarding the respiratory-visual manipulations. The study was performed in accordance with the Declaration of Helsinki and approved by the ethics research committee of the University of Lausanne, Switzerland. The subjects gave written informed consent to participate.

### Experimental methods

#### Full body illusion

The present protocol adapted a respiratory-visual manipulation experimental design previously used to identify the contribution of breathing to BSC^4^, within the frame of the full body illusion paradigm^5, 11, 17, 25, 40^. Participants comfortably lay on their back on a mattress placed on a table 75 cm above the ground. They wore an immersive virtual reality head-mounted display (Oculus Rift Development Kit 1, 640 × 800 resolution per eye, Oculus VR, Menlo Park, CA, USA) on which a photograph of a subject of the same built and gender (the avatar) taken from the back was displayed. The study participants therefore had the impression of viewing themselves from the back. Breathing activity was measured in real-time by a hand-held prototype from SmartCardia (EPFL Innovation Park, Lausanne, Switzerland) developed in collaboration with the Embedded Systems Laboratory at EPFL (http://esl.epfl.ch) that detected body impedance changes during breathing with four electrodes in contact with the subject’s fingers. This signal was processed online by an in-house virtual reality experimental design software (ExpyVR, http://lnco.epfl.ch/expyvr) that displayed a halo around the avatar and modulated its transparency either in synchrony or out of synchrony with the breathing cycle. The halo became increasingly visible during inspiration and reached a peak at the end of inspiration. Transparency was reset at the beginning of each inspiration.

#### Experimental induction of dyspnoea

**Figure 1:**
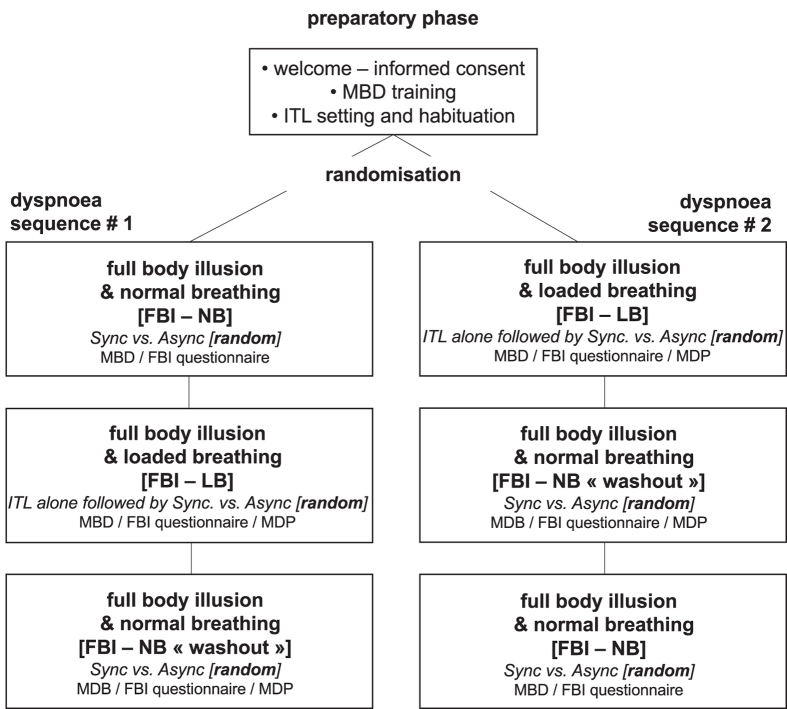
Flow chart of the experimental sequence. ITL, Inspiratory Threshold Loading; FBI, full body illusion; NB, Normal Breathing; LB, Loaded Breathing; Sync, synchronous respiratory-visual stimulation; Async, asynchronous respiratory-visual stimulation; MBD, mental ball drop task; MDP, Multidimensional Dyspnea Profile. The participants were randomised to participate either in “dyspnoea sequence 1” or in “dyspnoea sequence 2”. For each induction of full body illusion, the order of synchronous and asynchronous illumination was randomised.

Dyspnoea was induced through inspiratory threshold loading (Power breathe Classic, MT Technologies, Birmingham, UK). With such devices, the subject has to overcome an adjustable inspiratory pressure threshold before inspiratory flow can be produced, and to maintain the effort above the threshold to pursue inspiration. During the “habituation” step of the experimental sequence (see below and Fig. 1), the load was adjusted in such a way as to obtain a rating of breathing unpleasantness amounting to at least 6/10 on a 10 cm left-to-right visual analogue scale (VAS; A1 scale of the Multidimensional Dyspnea Profile, see below). To this aim, maximum inspiratory pressure (MIP) was first measured in each subject (Morgan MicroMPR, CareFusion, BD AG, Allschwill, Switzerland; median [IQR]: 87 cmH_2_O [69,110] in the study population) following standard procedures^41^. The initial threshold load was set to 60% of MIP (according to previous observations made in the laboratory using the same approach) and then adjusted in a staircase manner to reach a level of 6/10 on the A1 VAS. This loading level was subsequently used during all loaded breathing periods without further adjustment. Breathing frequency was not controlled. Loading levels were similar between men and women. Of note, we chose to determine the load based on dyspnoea unpleasantness in line with the study hypothesis (see introduction), because of its known relationship with insular activation^32, 35, 37^.

#### Assessment of Bodily Self-Consciousness

Bodily self-consciousness was assessed using an eight-statement questionnaire describing the putative changes in BSC induced by the respiratory-visual stimulation^4, 5^. The questionnaire explored different subjective feelings of BSC, namely the experienced location of breathing (Q1: *“It seemed as if I was feeling my respiration where the virtual body was”*), awareness of the respiratory-visual experiment manipulation (Q2: *“It seemed as if the flashing was my respiration”*), breathing agency (Q3: *“It seemed as if the virtual body was using my lungs to breathe”*; Q4: *“I felt as if the virtual body was breathing with me”*); self-identification with the virtual body (Q5: *“I felt as if the virtual body was my body”*). Three additional statements (Q6: *“I felt as if my real body was drifting towards the virtual body”*; Q7: *“I felt as if the virtual body was drifting towards my real body*”; Q8: *“It seemed as if I had two bodies”*) were also administered. These 3 items were considered as “control” insofar as they normally fail to reveal significant differences between synchronous and asynchronous stimulation during visuo-tactile, cardio-visual, and respiratory-visual experiments^4, 5, 14^. For each item, participants were asked to rate their agreement with the statements expressed in the question on a 7-point Likert scale with scores ranging from −3 (absolutely disagree) to +3 (absolutely agree).

In addition to the above subjective assessment, we used the mental ball drop task to measure the perceived self-location during the full body illusion^4, 17, 40^. In this task participants are asked to imagine dropping a ball from their hand (at the level of their body lying supine) to the floor and to evaluate the time spent by the ball to hit the floor. Participants press a button with their index finger when they imagine dropping the ball, keep the button pressed during the imagined fall, and release the button at the time of the imagined floor hit. The duration of button press (response time) has been shown to be a sensitive marker of self-location above the floor^4, 40, 42^. A longer reaction time indicates elevation of self-location (with respect to the floor) and therefore a mislocalisation towards the virtual body. Before the beginning of the experiment, participants performed a training session with at least 20 trials. During the experiments, three repeated mental ball drop tasks were performed at baseline and three repeated tasks were performed at the end of each experimental block.

#### Assessment of dyspnoea

Dyspnoea was characterised and quantified using the Multidimensional Dyspnea Profile^43^. In brief, the Multidimensional Dyspnea Profile consists of three subscales: Unpleasantness (A1), Sensory (S), and Affective (A2). The A1 subscale assesses the immediate unpleasantness of dyspnoea on a 0–10 visual numerical scale (from 0, “neutral”, to 10, “unbearable”): the higher A1 numeric values correspond to the more unpleasant breathing sensations. The sensory subscale includes five “sensory” items (“*my breathing requires work or effort*”; “*I am not getting enough air*”; “*my chest and lungs feel tight or constricted*”; “*my breathing requires mental effort or concentration*”; “*I am breathing a lot*”). Subjects are asked to choose the one that best describes their respiratory sensation and then to rate each of these descriptors on a 0–10 scale:: the sum of these ratings quantifying the sensory dimension of dyspnoea (the higher “S” values correspond to more intense breathing sensations). The affective subscale includes five affective items (“my breathing makes me depressed”, “anxious”, “frustrated”, “angry”, “afraid”). Subjects are asked to rate each of these descriptors on a 0–10 scale: the sum of these ratings quantifying the affective dimension of dyspnoea (the higher “A2” values correspond to stronger breathing-related negative emotions).

### Experimental sequence

Participants were first welcomed to the experimental room and briefed about the methods used to induce dyspnoea, the stimuli that they were going to experience, and the tasks that they would be asked to perform (full body illusion questionnaire, mental ball drop test). They were then familiarised with inspiratory threshold loading, both to determine the appropriate level of loading (see above, “experimental induction of dyspnoea”) and for “habituation” to minimise the impact of a discovery effect in the interpretation of the results. After this preparatory phase (Fig. 1), the participants were equipped with the immersive virtual reality device and installed in the supine position on the experimental table. They were randomised to undergo either “Sequence 1” or “Sequence 2”, as follows (Fig. 1). Sequence 1 started with a normal breathing block, with synchronous or asynchronous illumination of the avatar in random order (“full body illusion/normal breathing”). During a second block, inspiratory loading was first applied without any avatar illumination and the corresponding dyspnoeic response was evaluated (“loaded breathing without avatar illumination”). The effect of synchronous or asynchronous illumination of the avatar was then tested, in random order (“full body illusion/loaded breathing”), and dyspnoea was evaluated in both conditions of illumination. The subjects then resumed normal breathing and the evaluations were repeated (“washout”, see below). Sequence 2 started with the block comprising loaded “breathing without avatar illumination” and “full body illusion/loaded breathing”. This was followed by a washout block, and then by the full body illusion/normal breathing block. The washout block during sequence 2 was intended to verify the absence of carry-over effect from the full body illusion/loaded breathing to the full body illusion/normal breathing block. The corresponding data were not entered in the statistical analysis. The washout block during sequence 1 was performed for the sake of symmetry with sequence 2, and to verify the absence of long-lasting effects.The participants answered the full-body illusion questionnaire and performed the mental ball drop test at the end of each illumination period. They also answered the Multidimensional Dyspnea Profile at the end of each period involving inspiratory loading.

### Statistical analysis

The full body illusion questionnaire data and the Multidimensional Dyspnea Profile data were not normally distributed. The data from each question underwent an intra-subject standardisation by means of an ipsatisation procedure in order to neutralise the effect in responses set^44, 45^. Specifically, each rating was decreased by the mean rating of the subject responses to all questions and conditions and then divided by the standard deviation of subject’s responses to all questions and conditions. Ipsatisation therefore transformed questionnaire ratings in z-scores, of which the normal distribution allowed a proper use of parametric tests on questionnaire data^44–47^. Mental ball drop data were normally distributed and are therefore summarised as mean ± SD. As the response at the mental ball drop task is biased by each participant’s response criterion, we corrected each response for the previously assessed baseline (see Methods). To do so, we subtracted for each participant their average score for Baseline trials from the averaged score of mental ball drop trial performed at the end of the block.

For the full body illusion questionnaire, a repeated measure ANOVA was conducted on the ipsatised values separately for each question with two within subject factors: Breathing condition (NB -normal breathing-, LB -loaded breathing-) and Stimulation (Synchronous, Asynchronous). For the Multidimensional Dyspnea Profile, the ipsatised values were entered in a repeated measure ANOVA separately for the three different subscales (A1, S, A2) for the full body illusion/loaded breathing condition only, with the factor “stimulation” (no stimulation, synchronous, asynchronous). An additional ANOVA was performed to investigate any difference between the three breathing conditions (loaded breathing without avatar illumination, full body illusion/normal breathing, full body illusion/loaded breathing) in an “immediate perception domain” (obtained by summing the scores of the A1 and S subscales), as a standardised procedure in the analysis of the Multidimensional Dyspnea Profile questionnaire^27, 43, 48, 49^. Of note, and because gender affects the perception of loaded breathing^50^, we conducted similar analyses in the population restricted to the female participants.

For all statistical tests, a p value below 0.05 was considered significant. Significant interactions were explored by using Newman Keuls post-hoc tests, both for the full body illusion questionnaire and the Multidimensional Dyspnea Profile questionnaire”.

## Results

### Bodily self-consciousness

#### Full-body illusion questionnaire

**Figure 2:**
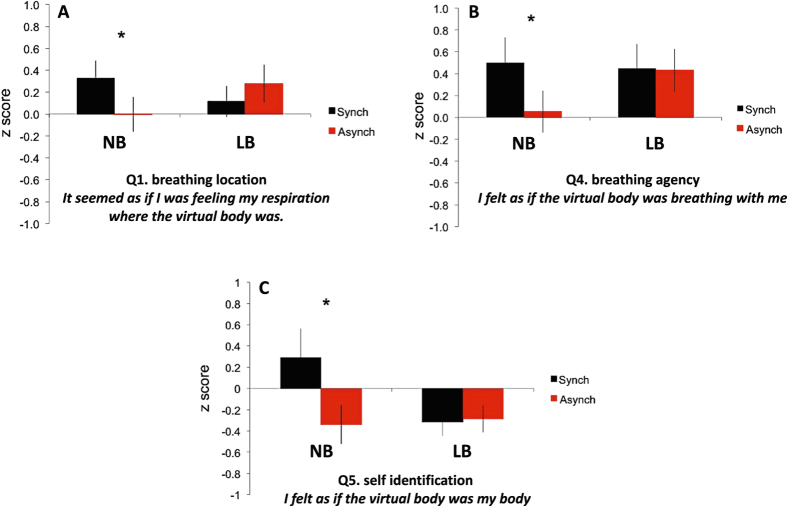
Effects of inspiratory threshold loading on Bodily Self-Consciousness. Mean questionnaire ratings, transformed in z-scores, for normal breathing (NB) and loaded breathing (LB), during synchronous and asynchronous respiratory-visual illumination. The error bars denote the standard error of the mean (SEM).

Results from the full body illusion questionnaire confirmed and extended previous results using a similar set–up^4^. Seeing the virtual body halo flashing in synchrony with their breathing affected participants’ perceived breathing location, but this depended on the breathing condition, as revealed by the significant two-way interaction [F(1,24) = 6.82, p = 0.02]. Indeed, as expected in the full body illusion/normal breathing condition, during synchronous respiratory-visual stimulation participants more strongly misperceived their breathing at the position of the virtual body (Q1 *“It seemed as if I was feeling my respiration where the virtual body was”*), as compared to the asynchronous control condition (Sync mean z-score = 0.39; Async mean z-score = 0.01; p = 0.04; Fig. 2A). Importantly, this synchronous-asynchronous difference was not observed in the LB condition (Sync = 0.12, Async = 0.27, p = 0.24).

Similarly, the synchrony-dependent modulation of breathing agency (Q4: *“I felt as if the virtual body was breathing with me”*) was also only observed in the NB condition, as revealed by a significant two-way interaction [F(1,24) = 5.42, p = 0.03]. Post-hoc testing revealed that during the full body illusion/normal breathing condition synchronous respiratory-visual stimulation altered breathing agency, resulting in a stronger feeling that the avatar was breathing with the participants as compared to asynchronous stimulation (Sync = 0.74, Async = −0.15, p = 0.03, Fig. 2B). This synchronous-asynchronous difference was absent during the LB condition (Sync mean z-score = 0.47; Async mean z-score = 0.40, p = 0.23), but the Q4 mean z-scores were positive during inspiratory threshold loading, compatible with some degree of illusory perception.

Synchronous respiratory-visual stimulation also resulted in higher illusory self-identification (Q5 “*I felt as if the virtual body was my body*”) as compared to asynchronous respiratory-visual stimulation (significant two-way interaction; F(1,24) = 6.17, p = 0.02), again only during the NB condition (NB: Sync = 0.22, Async = −0.36, p = 0.01, Fig. 2C; LB: Sync mean z-score = −0.30, Async mean z-score = −0.30, p = 0.51).

No statistically significant differences were found across conditions for any of the control (Q6, Q7, Q8) questions or the other experimental questions Q2, Q3.

#### Self-location (mental ball drop task)

The ANOVA on mean response time with the within-subject factors Breathing (Normal, Loaded) and Stimulation (Synchronous, Asynchronous) did not reveal any significant main effect or interaction (all p_s_ > 0.09). An additional ANOVA was also run on the raw response times (not corrected by Baseline), leading to the same null result (all p_s_ > 0.66)^51^.

### Dyspnoea – Multidimensional Dyspnea Profile results

**Figure 3:**
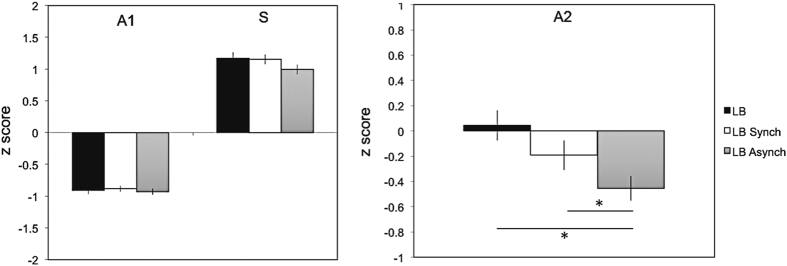
Effects of inspiratory threshold loading without and with respiratory-visual synchronous and asynchronous stimulation on the components of dyspnoea as explored by the Multidimensional Dyspnea Profile. LB, Loaded Breathing alone; LB Sync, Loaded Breathing Synchronous respiratory-visual illumination; LB Async, Loaded Breathing Asynchronous respiratory-visual stimulation; **A1**, immediate unpleasantness of dyspnoea; S, sensory dimension of dyspnoea; **A2**, affective component of dyspnoea (see “Methods” for details); the error bars denote the standard error of the mean (SEM); the * symbol denotes p < 0.05.

The detailed results of the Multidimensional Dyspnea Profile evaluation are provided in Table 1. Regarding the A1 scale, the S scale, and the A1 + S immediate perception domain, the values during “loaded breathing without avatar illumination”, “loaded breathing with synchronous respiratory-visual stimulation” and “loaded breathing with asynchronous respiratory-visual stimulation” were not significantly different (p = 0.32, 0.30 and 0.38, respectively (Fig. 3, left panel). In contrast, the A2 values during “loaded breathing with synchronous respiratory-visual stimulation” and “loaded breathing with asynchronous respiratory-visual stimulation” were numerically lower than during “loaded breathing without avatar illumination” (Fig. 3, right panel), suggesting a reduction in the intensity of the negative feelings associated with breathing when respiratory-visual stimulation was present. Statistical analysis confirmed a significant effect of respiratory-visual stimulation [F(2,48) = 7.09, p < 0.01]. Post-hoc testing revealed that participants reported significantly lower A2 scores during loaded breathing with asynchronous respiratory-visual stimulation than during loaded breathing without avatar illumination (mean z-score = −0.45 vs 0.04, respectively; p < 0.01). They also reported significantly lower A2 scores during loaded breathing with asynchronous respiratory-visual stimulation than during loaded breathing with synchronous respiratory-visual stimulation (mean z-score = −0.45 vs −019, respectively; p < 0.04). Although A2 scores during loaded breathing with synchronous respiratory-visual stimulation were lower than during loaded breathing without avatar illumination, this difference did not reach statistical significance (p = 0.10).

**Table 1:** Multidimensional Dyspnea Profile detailed results in the different conditions (median and interquartile range -IQR-).

	A1 score (unpleasantness of breathing)	Loaded breathing without avatar illumination	Loaded breathing with synchronous respiratory visual stimulation	Loaded breathing with asynchronous respiratory visual stimulation
		median [IQR]: 6.5 [4.3–8.0]	median [IQR]: 8.0 [5.0–8.0]	median [IQR]: 6.0 [5.3–8.0]
Sensory descriptors	My breathing requires muscle work or effort	apply 100%; best apply 19% median [IQR]: 8.0 [5.3–8.0]	apply 96%; best apply 15% median [IQR]: 8.0 [5.0–9.0]	apply 92%, best apply 31% median [IQR]: 6.5 [6.0–8.0]
	I am not getting enough air I am smothering I feel hunger for air	apply 100%; best apply 42% median [IQR]: 7.0 [6.0–9.0]	apply 96%; best apply 31% median [IQR]: 7.0 [5.3–9.0]	apply 96%; best apply 19% median [IQR]: 6.0 [5.0–8.0]
	My chest and lungs feel tight or constricted	apply 85%; best apply 4% median [IQR]: 5.5 [2.0–7.0]	apply 88%; best apply 4% median [IQR]: 6.0 [1.0–7.8]	apply 88%; best apply 8% median [IQR]: 5.0 [1.5–6.8]
	My breathing requires mental effort or concentration	apply 100%; best apply 35% median [IQR]: 8.0 [7.0–9.0]	apply 100%; best apply 50% median [IQR]: 8.0 [6.3–9.0]	apply 96%; best apply 34% median [IQR]: 7.0 [7.0–9.0]
	I am breathing a lot	apply 92%; best apply 0% median [IQR]: 5.5 [5.0–8.0]	apply 96%; best apply 0% median [IQR]: 6.0 [2.8–8.0]	apply 96%; best apply 8% median [IQR]: 6.0 [2.5–8.0]
	**S score**	**median [IQR]: 33.0 [30.0–38.0]**	**median [IQR]: 32.0 [25.3–40.5]**	**median [IQR]: 28.5 [25.3–36.3]**
affective descriptors	My breathing makes me feel:			
	depressed	apply 35% median [IQR]: 0.0 [0.0–2.8]	apply 38% median [IQR]: 0.0 [0.0–1.0]	apply 31% median [IQR]: 0.0 [0.0–1.0]
	anxious	apply 92% median [IQR]: 7.5 [6.3–9.0]	apply 92% median [IQR]: 6.0 [2.3–8.0]	apply 100% median [IQR]: 6.0 [3.0–7.0]
	frustrated	apply 77% median [IQR]: 5.0 [2.0–6.8]	apply 73% median [IQR]: 3.0 [0.3–6.0]	apply 65% median [IQR]: 1.5 [0.0–5.8]
	angry	apply 31% median [IQR]: 0.0 [0.0–2.0]	apply 35% median [IQR]: 0.0 [0.0–2.0]	apply 35% median [IQR]: 0.0 [0.0–1.8]
	afraid	apply 81% median [IQR]: 5.5 [1.3–8.0]	apply 81% median [IQR]: 3.5 [1.0–6.8]	apply 73%median [IQR]: 2.0 [0.3–5.0]
	**A2 Score**	**median [IQR]: 19.0 [11.8–24.5]**	**median [IQR]: 14.0 [5.8–20.8]**	**median [IQR]: 12.0 [5.3–17.5]**

In the “sensory descriptors” cells, the first line gives the percentage of cases where the participants described the corresponding descriptor as applying to the respiratory sensations they experienced during loaded breathing; this is followed by the indication of the percentage of cases were this descriptor was chosen as the best one by the subjects. In the “affective descriptors” cells, the first line gives the percentage of cases where the participants described the corresponding descriptor as applying to the emotions that they felt during loaded breathing. In all these cells, the second line provides the median and interquartile range of the scoring of the corresponding descriptor in the study population.

### Effects of gender

Restricting the analysis to the female component of the study population did not modify the pattern of responses to the full body illusion questionnaire or the Multidimensional Dyspnea Profile, nor did it modify the significant differences observed in the complete population. The results from the mental ball drop task were not significant either.

## Discussion

This study corroborates and extends previous findings regarding the contribution of breathing to BSC in healthy humans^4^. It reports the new finding that experimental dyspnoea in healthy subjects disrupts the full body illusion (Fig. 2). It also reports the new finding that respiratory-visual stimulation reduces the intensity of negative emotions associated with dyspnoea (A2 score of the Multidimensional Dyspnea Profile) (Fig. 3).

### Corroboration of the contribution of breathing to bodily self-consciousness

In this study, we observed that synchronous visual illumination of a virtual body with respect to one’s own inspiration resulted in a mislocalisation of breathing towards the virtual body, a transfer of breathing agency to the virtual body, and an illusory self-identification with the virtual body. These changes in breathing location toward a virtual body and in breathing agency replicate and corroborate previous data^4^. The two studies thus converge to substantiate the contribution of breathing to BSC. This complements data showing the contribution of cardiac signals to BSC^5, 6, 11, 16, 23, 25, 52^. The data obtained during normal breathing in our subjects thus strengthen the hypothesis that interoceptive signals are processed jointly with exteroceptive signals to build bodily self-consciousness^6, 8, 13^ and awareness of body environment^24^. In contrast with previous work^4^, we did not see any effect on the mental ball drop task. We do not have a firm explanation for this, but we note that incongruences between this test and the results of questionnaires have been described before.

### Dyspnoea abolishes the effects of respiratory-visual stimulation on bodily self-consciousness

Several non-mutually exclusive mechanisms may be associated with the cancelling effect of inspiratory threshold loading on the full body illusion that we observed. These are (1) attentional and emotional effects; (2) imbalance between the intensities of the various sensory inputs to the brain; and (3) competition for cortical resources.

Firstly, attentional and emotional effects are strong candidates to explain our observations. Both could simply have diverted our subjects from the virtual body that they were looking at. However, in other studies relying on interference paradigms rather similar to the one we used (i.e. the counter-irritation phenomenon where a pre-existent pain is attenuated by the heterotopic application of a novel noxious stimulus) it has been shown that distraction is not sufficient to fully explain the observed changes^53^.

Secondly, under normal conditions the respiratory signals that contribute to BSC are of low intensity and do not give rise to conscious perception^1^. In this study, inspiratory loading induced somatic awareness of breathing and gave a noxious dimension to respiration^54–58^ with a strong aversive emotional charge^59–62^. These changes in the nature of the respiratory afferent information may have modified the balance between the inputs to the brain that contribute to bodily self-consciousness in such a way as to render synchronous respiratory-visual stimulations inefficient at producing illusory effects. In this regard, previous work has shown that painful stimuli can abolish or decrease illusory hand ownership^63^. The inspiratory loading effect that we observed is also reminiscent of the findings reported by Palluel *et al*.^64^ where leg muscle vibrations applied during a full body illusion experiment cancelled the effects of synchronous visuo-tactile stimulation on BSC. Of note, dyspnoea is an aversive and threatening experience that induces behaviours aimed at its relief ^65, 66^. From a teleological perspective, our findings that dyspnoea abolishes illusory self-identification to an external avatar could be metaphorically interpreted as “I cannot leave this body that is under threat”, dyspnoea needing to be the foremost focus above all other considerations^65^. Another interpretation could be “I must focus all my attention on this body that is under threat” (see above). The decreased in the A2 component of the Multidimensional Dyspnea Profile that was induced by respiratory-visual stimulation (Fig. 3; see below) seems to support this reasoning.

Thirdly, the cortical networks involved in BSC and in dyspnoea share several key anatomical regions (most prominently, the supplementary motor area and the insula, with a potential right lateralisation in both cases)^13, 18, 31, 32, 37^. Neural interferences within this partially shared network could therefore contribute to explaining our observations. In this regard, we chose to manipulate inspiratory threshold loading as it activates both the supplementary motor area and the insula^31^, in contrast with carbon dioxide retention experiments that increase limbic activity without premotor activity)^30, 32, 33^. That dyspnoea interferes with BSC falls in line with dyspnoea-related disruptions of other motor or cognitive processes^38, 39^. For example, Nierat *et al*.^38^ reported that inspiratory threshold loading altered the ability of normal subjects to perform a sequential motor task, as during other dual-tasking paradigms. Sharman *et al*.^39^ described a patient suffering from congenital central alveolar hypoventilation in whom suppressing respiratory-related cortical activation^67^ through mechanical ventilation improved cognitive performances.

### Effects of respiratory-visual stimulations on dyspnoea

Respiratory-visual stimulations during inspiratory threshold loading did not induce the full body illusion. However, both synchronous and asynchronous respiratory visual stimulations significantly reduced the A2 component of the Multidimensional Dyspnea Profile (Table 1, Fig. 3). In other words, they decreased the intensity of the negative emotions associated with dyspnoea. Dyspnoea bears many similarities with pain^54, 55, 57, 65, 68–71^. Yet several studies have shown that visuo-tactile stimulation can decrease the perception of pain and related physiological responses^46, 72, 73^. In these studies, the alleviation of pain was associated with illusory self-identification with the virtual body, leading to the idea that illusory self-identification with a healthy virtual body may reduce pain perception. In the present study, respiratory-visual stimulations did not modify BSC during inspiratory threshold loading (Fig. 2). An alternative explanation is therefore needed. A recent imaging study revealed that synchronous interoceptive (cardiac)-visual stimulation induced insular activation that was further enhanced by asynchronous interoceptive(cardiac)-visual stimulation^74^. We propose that our findings of a decreased A2 intensity during respiratory-visual stimulation could stem from competitive insula activation by respiratory-visual stimulation and by inspiratory threshold loading^31, 37^. A similar hypothesis has been made to explain the interference between dyspnoea induced by carbon dioxide retention and cortical potentials evoked by painful laser skin stimulation, the source of which includes the anterior insula^75, 76^.

### Study limitations and merits — Conclusions

This study, although it corroborates earlier respiratory-visual findings on BSC and brings novel information about the interactions of breathing and BSC, is preliminary in nature. It raises many questions. It also has a number of limitations. Among these, there is one major difference between our experiments and the other full body illusion experiments. In our case, the multisensory manipulation included the very system on which the effects were studied. In contrast, in previous full body illusion experiments on pain^72, 73^, the multisensory manipulation and the outcome of interest (visuo-tactile stimulation and pain, respectively) were orthogonal, concerning different systems. It will thus be important to compare the present effects with potential full body illusion effects induced by visuo-tactile stimulation on dyspnoea. Another limitation of our study lies in the fact that it concerns only one type of experimental dyspnoea in healthy subjects. It will thus be necessary to conduct similar experiments with other types of experimental dyspnoea (e.g. dyspnoea induced by carbon dioxide retention and chest restriction).

In spite of these limitations, the main merit of this study is to open two new angles under which chronic respiratory diseases should be considered. One of these regards how chronic dyspnoea could alter BSC in patients with chronic respiratory diseases, and whether or not this has an impact on their quality of life. The other angle regards the investigation of the potential of medical applications of respiratory-visual stimulations as a method to alleviate dyspnoea in chronic respiratory diseases. The ability of this approach to mitigate the affective dimension of dyspnoea irrespectively of sensory changes (note that only the latter has so far been reported to be impacted by pharmacological interventions^77^, pulmonary rehabilitation^78–80^, and cognitive-behavioural strategies^81^) is particularly important in the numerous conditions were dyspnoea persists in spite of maximal “sensory-oriented” treatment of the underlying condition (“chronic breathlessness syndrome”^82, 83^; or when no such treatment is available.
